# Exploration of a nomogram prediction model of 30-day survival in adult ECMO patients

**DOI:** 10.3389/fmed.2023.1062918

**Published:** 2023-02-28

**Authors:** Liangwen Cui, Yutao Zha, Cheng Zhang, Hui Zhang, Chao Yu, Huang Rui, Min Shao, Nian Liu

**Affiliations:** ^1^Department of Critical Care Medicine, The First Affiliated Hospital of Anhui Medical University, Hefei, China; ^2^Department of Anhui Provincial Cancer Institute, The First Affiliated Hospital of Anhui Medical University, Hefei, China; ^3^Anhui Maternal and Child Health Hospital, Hefei, China

**Keywords:** extracorporeal membrane oxygenation, critical care medicine, nomogram model, prognosis, survival

## Abstract

**Objective:**

To investigate the factors of 30-day survival in ECMO patients, establish a nomogram model, and evaluate the predictive value of the model.

**Methods:**

A total of 105 patients with extracorporeal membrane oxygenation (ECMO) were admitted to the Department of Critical Care Medicine, The First Affiliated Hospital of Anhui Medical University, from January 2018 to March 2021. Cox regression analysis screened out the risk factors. Based on the results of multivariate analysis, the nomogram model was established by using R software, and the discrimination of the model was verified by bootstrap and calibration.

**Results:**

The results showed that sex, acute physiology and chronic health evaluation (APACHE) II score, disseminated intravascular coagulation (DIC) score before ECMO initiation and average daily dose of norepinephrine were independent risk factors for prognosis. Verify that the nomogram model is verified by bootstrap internally, and the corrected C-index is C-index: 0.886, showing a good degree of discrimination. The calibration curve (calibration) showed that the nomogram model had good agreement. The decision curve analysis(DCA) curve shows good clinical validity above the two extreme curves. Kaplan–Meier curves were drawn for patients in the tertile and compared with the first and second groups. The third group predicted the worst 30-day prognosis for ECMO patients.

**Conclusion:**

The nomogram prediction model constructed based on the sex, APACHE II and DIC score, average daily dose of norepinephrine can effectively screen out the factors affecting the prognosis and provide a reference for individualized treatment of ECMO patients.

## Introduction

Refractory respiratory and/or cardiogenic failure are the most critical cases in the Department of Critical Care, with higher mortality. Extracorporeal membrane oxygenation (ECMO) has been used exponentially increasing over the last decade and is considered a lifesaving modality (1). However, in some cases, there were no benefits if the primary diseases were not reversible or there was no therapeutic reactivity. Certain conditions, such as severe neurologic injury, have a poor prognosis and may warrant discussion about weaning ECMO (2). It is necessary to perform risk stratification for ECMO and make more efficient decisions.

A nomogram is a popular prognostic tool with the ability to predict clinical events by integrating potential risk factors (3). Nomograms have been widely used for tumor prognosis (4–6) and have been effectively used to predict short-term and long-term survival for asymptomatic adults undergoing screening for cardiac risk factors (7). Thus, we hypothesized that a nomogram may also be feasible for the risk stratification of critically ill patients undergoing ECMO. These predictors will enable risk stratification, guide interventional studies, and optimize the allocation of limited human and technical resources in ECMO management.

The Respiratory ECMO Survival Prediction (RESP) score (8) and veno-arterial-ECMO (SAVE)-score (9) are relevant and validated tools to predict survival for patients undergoing ECMO. However, the general prediction model for the prognosis of ECMO patients maybe still requires further study. The purpose of this study was to explore the factors for 30-day survival and establish a nomogram model to predict survival.

## Methods

### Patients

From January 2018 to March 2021, a total of 105 adult patients underwent ECMO at the First Affiliated Hospital of Anhui Medical University, People’s Republic of China. The indications for ECMO followed the Extracorporeal Life Support Organization (ELSO) guidelines (10). VA-ECMO is the treatment of choice for various patients with acute biventricular failure, including acute myocardial infarction, fulminant myocarditis, sudden cardiac intervention, awaiting cardiac transplantation, acute right heart failure: acute massive pulmonary embolism and intractable ventricular arrhythmias. VV-ECMO is the treatment of choice for patients with acute respiratory failure due to various causes. The main indications include severe acute respiratory failure due to ARDS patients, lung transplant patients, bronchial asthma, pulmonary embolism, atmospheric airway obstruction, and other causes. This study’s exclusion criteria were as follows: (1) age < 18 years; (2) ECMO duration<2 days or > 28 days; and (3) bridge to heart or lung transplant; (4) periprocedural support for large airway stenosis.

**Figure 1:**
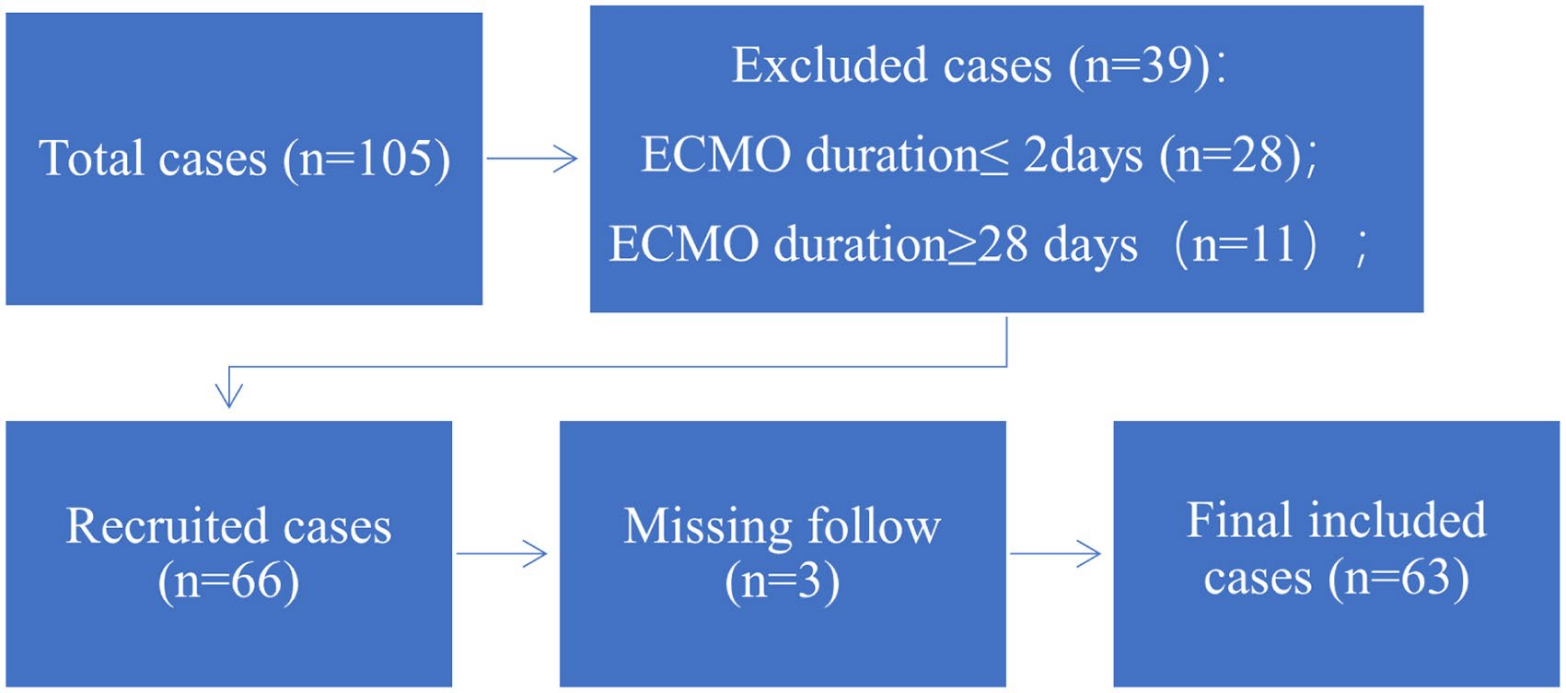
Flow chart.

According to the exclusion criteria, 63 patients were recruited for this study (Figure 1). Baseline characteristics, laboratory parameters, ECMO parameters, and in-hospital outcomes were reviewed from medical records. This was a retrospective cohort study in accordance with the Ethical Guideline of the Committee on Human Experimentation of our institution. Due to the nature of this retrospective study, informed consent was waived.

### Outcomes

The primary outcome was 30-day survival. The secondary outcomes included survival to ECMO weaning, ECMO duration, kidney injuries accepted continuous renal replacement therapy (CRRT), and ECMO-associated complications (leg ischemia, hospital acquired infections, thrombosis or hemorrhage).

### Statistical analysis

Continuous variables are expressed as the mean ± SD or median (IQR), as appropriate. Categorical data are expressed as numbers (percentages). Continuous variables were compared using Student’s *t* test or the rank-sum test, as appropriate. Categorical variables were compared by the *χ^2^* test. Univariate Cox regression was used to screen for variables that were significantly associated with 30-day survival in the primary cohort. The proportional hazards assumption was checked based on the scaled Schoenfeld residuals using the survival package in the R tool. Potential prognostic factors that were significant in the univariate Cox regression model were entered into the multivariate Cox proportional hazard model, in which the HR, which was used to approximate the risk of an event, was also calculated. To avoid too many variables entering into the final model and influencing the practicality of the model, a strict cut-off value of 0.05 was chosen. The backwards stepwise process based on the Akaike information criterion was used to control the overfitting of the model.

A two-tailed *p* value <0.05 was considered statistically significant in our study. SPSS software (V.26.0, IBM, New York, USA) and R software (V.3.6.3, R Foundation for Statistical Computing, Vienna, Austria) were used for statistical analysis.

### Nomogram analysis process

A nomogram based on the results of previous multivariable analyzes was established. The calibration, discrimination and clinical usefulness of the nomogram were calculated to evaluate its performance. The area under the receiver operating characteristic curve (AUC) and Harrell’s concordance index (C-index) were used to assess the predictive capacity of the prediction model (11). The calibration curve was used to analyze the agreement between the nomogram and actual observation. Decision curve analysis was performed to assess the clinical usefulness of the prognostic nomogram by quantifying the standardized net benefits at different threshold probabilities. We plotted Kaplan–Meier curves over the tertile of patients stratified by the scores predicted by the nomograms in the data set to further assess calibration. The model was validated using bootstrapped resampling to quantify any overfitting. Survival curves were used to compare the survival probability between the survival group and the non-survival group defined by the nomogram.

## Results

### Patient characteristics

This study used multiple interpolations to fill in the missing data (Supplementary Table S1). A total of 63 patients who underwent ECMO support were included in the study and divided into the survival group and non-survival group. There were 44 (69.84%) males and 19 (30.16%) females who received 28 venoarterial (VA) ECMO and 35 venovenous (VV) ECMO. The demographic and clinical characteristics were shown in Table 1. The age, height, weight, patient source, and comorbidities were not statistically significant between the two groups, and also no statistically significant between the VV-ECMO and VA-ECMO groups (*P>*0.05) (Supplementary Table S2). Clinical and laboratory data are shown in Table 2. Acute Physiology and Chronic Health Evaluation II (APACHE II) and sequential organ failure assessment (SOFA) score before ECMO initiation, mean daily dose of noradrenaline (NE), mean arterial pressure (MAP) and disseminated intravascular coagulation (DIC) score were statistically significant between the two groups (*p* < 0.05).

**Table 1:** Baseline characteristics of the included patients.

Variable	Total (*n* = 63)	Survival (*n* = 33)	Non-survival (*n* = 30)	*t/Z/x^2^ value*	*p*-value
Age (year)	53.02 ± 16.02	50.12 ± 14.53	56.2 ± 17.19	1.520	0.134
Sex(male)	44(69.84%)	20(60.61%)	24(80.00%)	0.001	0.9755
Height (cm)	167.68 ± 7.50	166.67 ± 7.84	168.80 ± 7.03	1.130	0.263
Weight (kg)	65.95 ± 9.38	65.09 ± 9.40	66.9 ± 9.42	0.762	0.449
Residence urban (*n*, %)	35(55.56%)	18(54.55%)	17(56.675)	0.029	0.866
Patient sources					
Inside-hospital (*n*)	27(42.86%)	15(45.45%)	12(40.00%)	0.033	0.856
Peripheral hospitals (*n*)	36(57.14%)	18(54.55%)	18(60.00%)	0.033	0.856
Comorbidity (*n*, %)					
Hypertension (*n*)	16(25.40%)	10(30.30%)	6(20.00%)	0.421	0.517
Diabetes(*n*)	7(11.11%)	5(15.15%)	2(6.67%)	0.447	0.504
Solid tumors(*n*)	9(14.29%)	4(12.12%)	5(16.67%)	0.024	0.877
Chronic respiratory disease (*n*)	4(6.35%)	0(0%)	4(6.25%)	2.724	0.989
Chronic cardiac disease (*n*)	12(19.05%)	7(21.21%)	5(16.67%)	0.019	0.891
Renal diseases (*n*)	4(6.35%)	1(3.03%)	3(10.00%)	0.380	0.538
Thyroid disease (*n*)	1(1.59%)	1(3.03%)	0(0%)	0.000	1.000
Nervous system diseases (*n*)	3(4.76%)	1(3.03%)	2(6.77%)	0.007	0.933
Autoimmune Disease (*n*)	2(3.17%)	1(3.03%)	1(3.33%)	0.000	1.000
Temperature (°*C*)	37.27 ± 0.76	37.25 ± 0.83	37.29 ± 0.70	0.198	0.844
HR (*n*)	110.95 ± 20.00	109.94 ± 27.62	112.06 ± 30.89	0.289	0.774
MAP (mmHg)	67.20 ± 12.82	70.36 ± 10.96	63.72 ± 13.95	−2.112	0.039
CVP (cmH2O)	14.22 ± 4.15	14.09 ± 4.44	14.36 ± 3.87	0.262	0.795
Laboratory parameters					
PT(*s*)	21.74(11.50,82.30)	23.56(12.80，82.30)	19.13(11.50,32.20)	−0.71	0.944
APTT(*s*)	49.03(27.50,140.60)	51.70(28,90,140.60)	44.9(27.50,63.10)	−1.085	0.288
CKMB(U/L)	117.40(3.00,1176.00)	139.40(3.00,1176.00)	83.60(3.00,501.00)	−0.753	0.457
cTn-I (ng/mL)	3.706(0.001,30.00)	2.86(0.010,28.84)	5.12(0.11,30.00)	−0.416	0.678
PCT (ng/mL)	18.85(0.06,169.00)	28.16(0.06,169.00)	7.20(0.06,33.95)	−0.494	0.626
BUN (mmol/L)	12.44(2.60,28.00)	11.12(2.60,24.90)	14.13(7.00,28.00)	−1.629	0.103
sCr (mmol/L)	156.51(35.50,366.40)	128.81(42.10,366.40)	191.90(35.50,361.00)	−1.655	0.098
TBIL (mmol/L)	25.34(6.50,91.00)	21.91(7.07,91.00)	29.98(6.50,77.40)	−1.382	0.503
ALT (U/L)	457.00(7.00,3743.00)	423.17(7.00,3174.00)	500.22(17.00,3743.00)	−1.038	0.229
AST (U/L)	738.54(39.00,696.00)	566.91(33.00,5559.00)	970.71(21.00,6124.00)	−0.670	0.503
PLT (10^9/L)	166.63(26.0,429.0)	179.83(73.0,429.0)	146.81(26.00,295.00)	−1.215	0.233
pH	7.27(6.87,7.54)	7.29(6.90,7.54)	7.25(6.87,7.52)	−0.686	0.493
PO_2_ (mmHg)	99.27(25.30,440.00)	97.67(45.10,247.00)	102.2(25.3,440.0)	−0.900	0.929
PCO_2_ (mmHg)	45.32(19.00,150.00)	43.71(19.00,72.00)	48.28(21.00,150.00)	−0.487	0.626
HCO_3_^−^ (mmol/L)	21.00(16.45,27.00)	20.00(17.25,26.38)	22.00(14.90,27.60)	−0.287	0.774
PO_2_/FIO_2_ (mmHg)	82.5(56.20,100.00)	82.5(61.91,198.84)	81.27(48.65,194.33)	−0.585	0.559
Lac (mmol/L)	3.80(1.85,7.30)	3.80(2.59,5.55)	3.53(1.74,9.73)	−0.206	0.836
ECMO type(*n*)					
V-A	28(44.44%)	14(42.42%)	14(46.67%)	0.007	0.933
V-V	35(55.56%)	19(57.58%)	16(53.33%)	0.000	1.000
Steroid (*n*)	28(44.44%)	14(42.42%)	14(46.67%)	0.007	0.933
CPR (*n*)	8(12.70%)	5(15.15%)	3(10.0%)	0.055	0.815
The daily average dose of NE (ug/kg/min)	1.28 ± 0.74	0.75 ± 0.42	1.86 ± 0.55	9.094	0.000
APACHE II	24.00(7.00,44.00)	17.00(7.00,29.00)	33.00(20.00,44.00)	−6.552	0.000
SOFA	11.00(5.00,19.00)	10.00(5.00,14.00)	12.00(6.00,19.00)	−2.032	0.042
DIC	3.00(1.00,4.00)	1.00(0.00,2.00)	4.00(4.00,5.00)	−6.201	0.000

HR, heart rate; MAP, mean arterial pressure; CVP, central venous pressure; PT, prothrombin time; APTT, activated partial thromboplastin time; CKMB, creatine kinase isoenzymes; cTn-I, cardiac troponin I; PCT, procalcitonin; BUN, Blood Urea Nitrogen; sCr, serum creatinine; TBIL, total bilirubin; ALT, alanine aminotransferase; AST, aspartate aminotransferase; PLT, platelet; PO2, Arterial partial pressure of oxygen; PCO2, Arterial blood carbon dioxide partial pressure; HCO3-, bicarbonate; PO2/FIO2,Oxygenation index; Lac, lactate; V-A, venoarterial; V-V, venovenous; CPR, cardiopulmonary resuscitation; NE, norepinephrine; APACHE II, acute physiology and chronic health evaluation II; SOFA, sequential organ failure assessment; DIC, disseminated intravascular coagulation.

**Table 2:** Comparison of the secondary outcomes between two groups.

Secondary outcome	Total (*n* = 63)	Survival (*n* = 33)	Non-survival (*n* = 30)	t/Z/x2 value	*P*-value
ECMO duration (hour)	158.38(107.875,239.00)	124.00(96.00,190.05)	191.25(126.50,296.18)	−0.2381	0.017
Total hospital stay (hour)	528.00(288.00,744.00)	672.00(324.00,864.00)	360.00(192.00,606.00)	−3.208	0.001
ICU stay (hour)	360.00(264.00,620.00)	408.00(288.00,715.00)	324.00(186.00,510.00)	−2.713	0.007
ECMO weaning (*n*)	46(73.02%)	33(100%)	14(46.67%)	20.861	0.000
CRRT (*n*)^1^	25(39.68%)	8(24.24%)	17(56.67%)	5.614	0.018
MV duration (hour)	292.50(169.00,472.00)	247.50(173.65,513.75)	303.00(158.60,422.70)	−0.048	0.962
Complication					
Thrombosis (*n*)	5(7.94%)	2(6.06%)	3(10.00%)	0.012	0.912
Bleeding (*n*)	6(9.52%)	1(3.03%)	5(16.67%)	1.993	0.158
pneumothorax (*n*)	2(3.17%)	0(0%)	2(6.67%)	0.621	0.431
HAIs(*n*)^3^	33(52.38%)	19(57.58%)	14(46.67%)	0.376	0.540

ECMO, Extracorporeal membrane oxygenation; CRRT, Continuous Renal Replacement Therapy; ICU, intensive care unit; MV, mechanical ventilation; HAIs, hospital acquired infections.

The secondary outcomes, the ECMO duration, length of total hospitalization, length of ICU stay, ECMO weaning, and CRRT were statistically significant between the two groups (*p <* 0.05) (Table 2).

**Figure 2:**
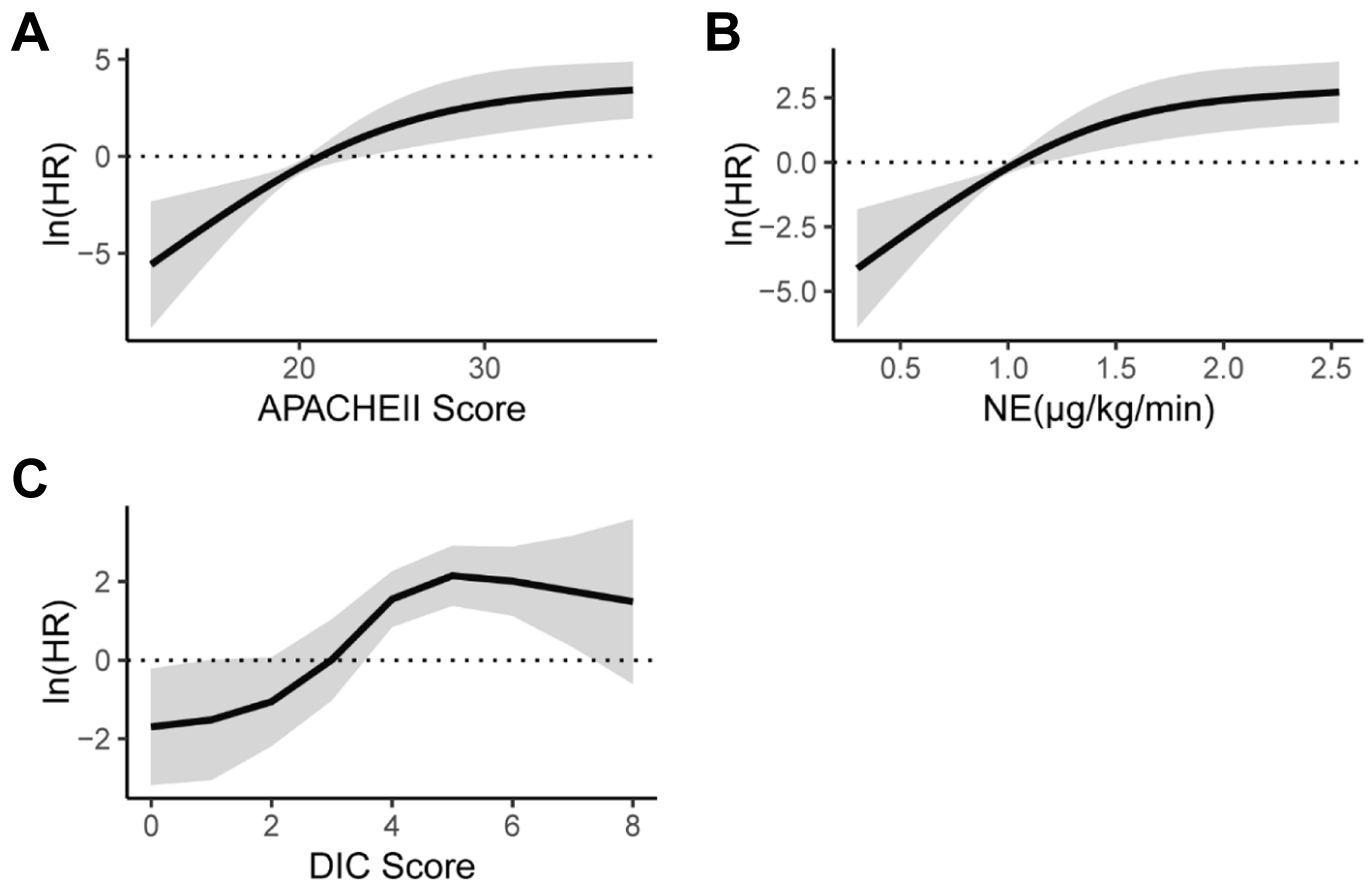
Nonlinear trend.

In univariate analysis, the APACHE II score before ECMO initiation, average daily dose of norepinephrine, and DIC score were statistically significant. In a nonlinear trend (Figure 2), within 30 of the APACHE II score before ECMO initiation, with the score increased, the mortality risk gradually increased; when the APACHE II score was greater than 30, the mortality risk became stable with the score increased (Figure 2A). When the average daily dose of NE was within 1.5 μg/kg/min, the mortality risk gradually with the dose increased; then the average daily dose of NE was greater than 1.5 μg/kg/min, the mortality risk tended to increase with increasing dose (Figure 2B). When the DIC score is within 4, as the score increases, the mortality risk gradually increases; when the DIC score is greater than 4, the mortality risk tends to be stabilized with the score increased (Figure 2C).

Because of the APACHE II score, so the age was not included separately in the multivariate analysis. Multiple epidemiologic studies have observed sex-related differences in admission into the ICU and ICU resources (12, 13). The statistical indicators and sex in the univariate analysis were incorporated into the Cox analysis. The results showed that the Sex, APACHE II and DIC score before ECMO initiation, average daily dose of NE were independent risk factors for prognosis (Table 3).

**Table 3:** Multivariate Cox regression analysis of prognosis related factors.

Variable	*Β*	SE	Wals-value	*P*-value	HR value	95% CI
Sex	1.207	0.581	4.321	0.038	3.345	1.071–10.440
SOFA	−0.012	0.071	0.029	0.865	0.988	0.861–1.135
APACHE II	0.114	0.042	7.529	0.006	1.121	1.033–1.216
DIC	1.372	0.714	3.694	0.055	0.254	0.063–1.027
The daily average dose of NE (ug/kg/min)	1.586	0.545	8.482	0.004	4.883	1.680–14.196
MAP (mmHg)	−0.021	0.020	1.124	0.289	0.979	0.942–1.018

NE, norepinephrine; APACHE II, acute physiology and chronic health evaluation II; SOFA, sequential organ failure assessment; DIC, disseminated intravascular coagulation; MAP, mean arterial pressure.

**Figure 3:**
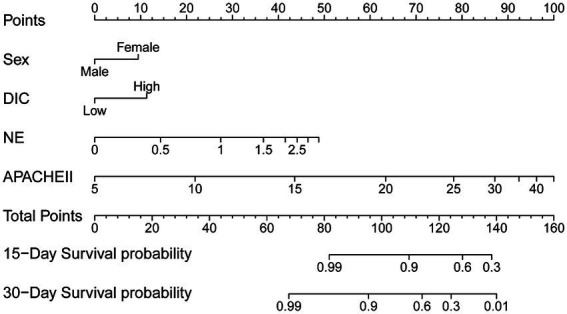
Nomogram chart.

**Figure 4:**
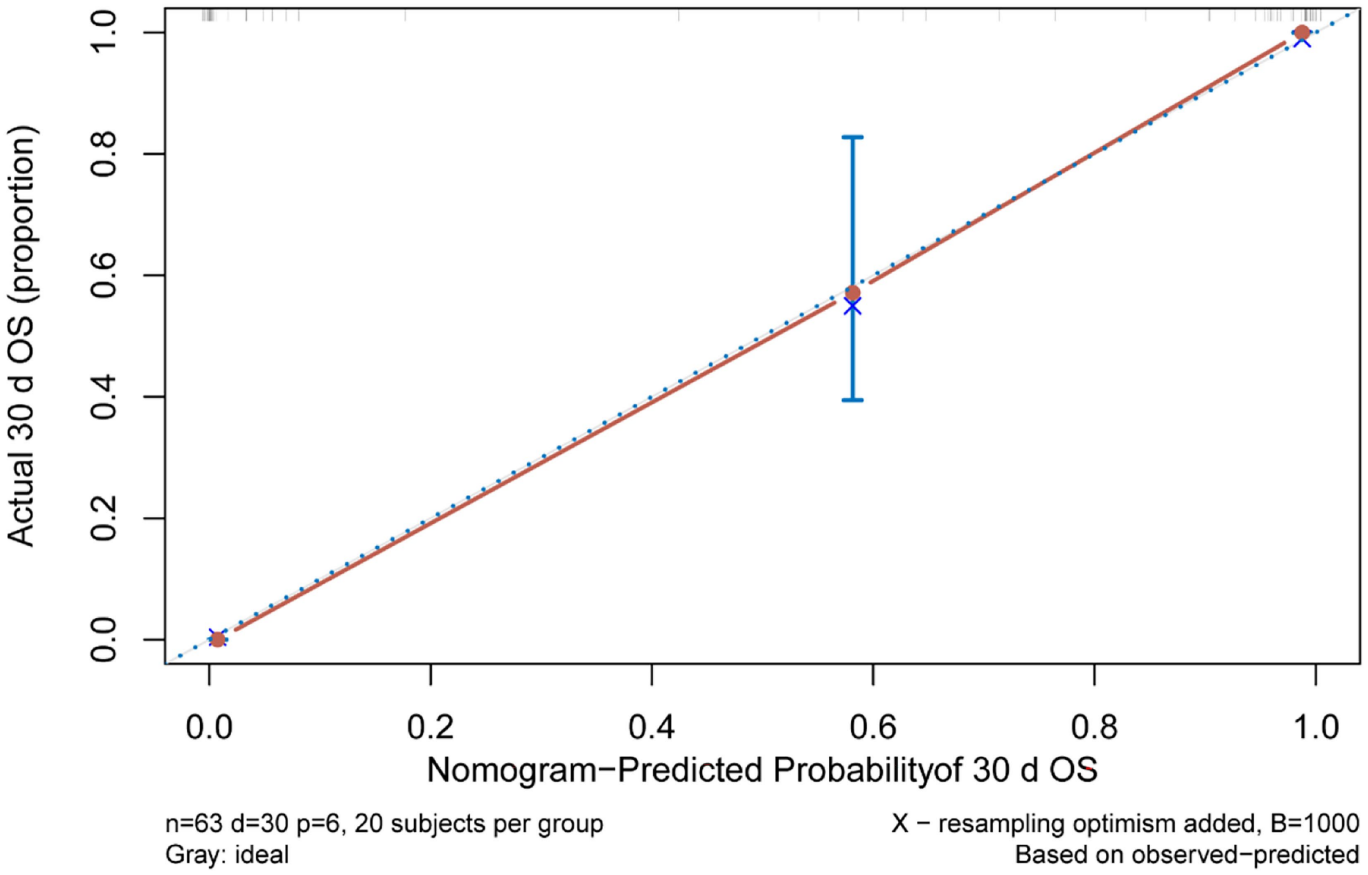
Calibration curve.

**Figure 5:**
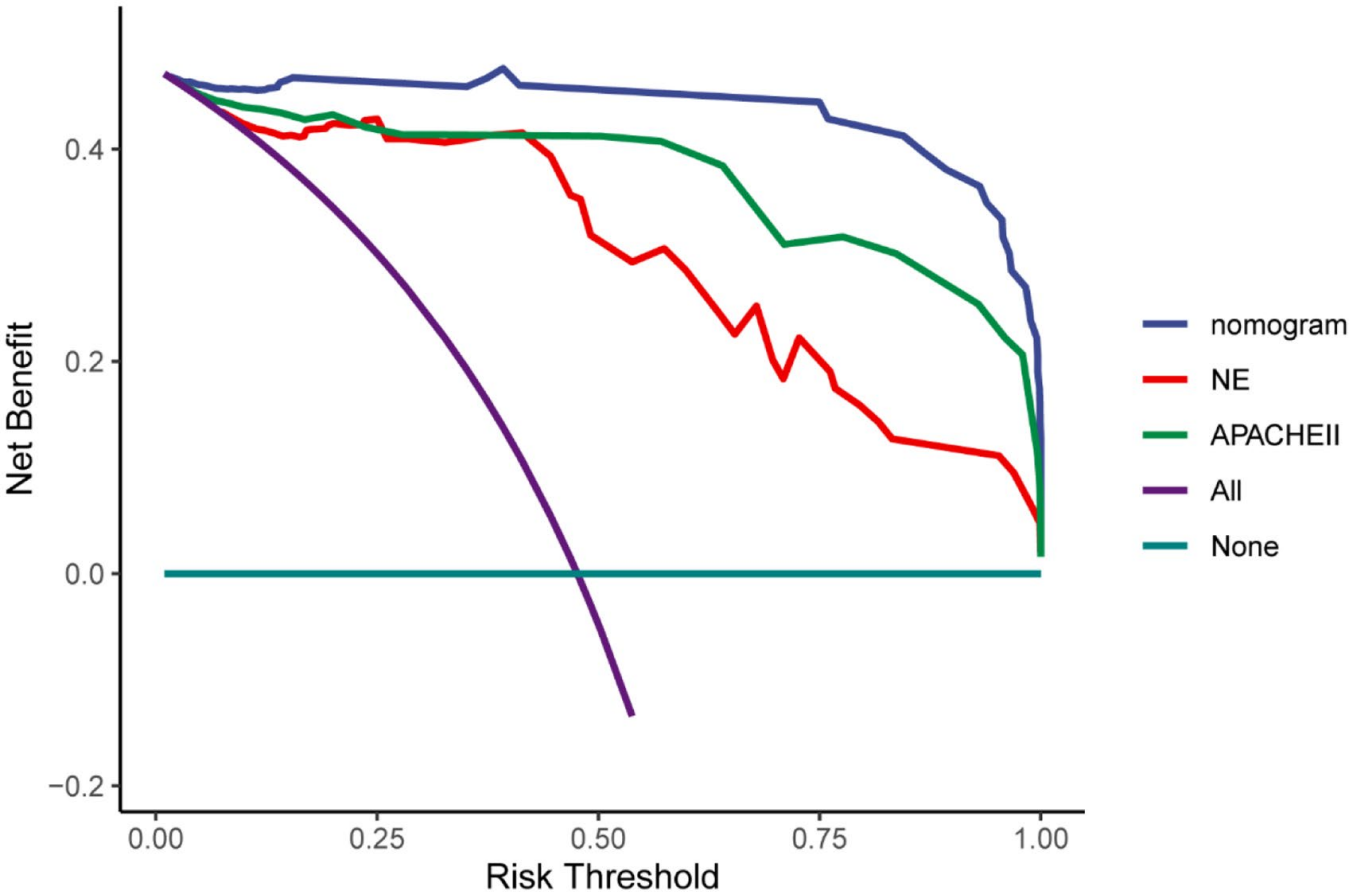
DCA curve.

Multivariate Cox regression analysis was used to screen out the risk factors of prognosis, and was selected to establish a nomogram chart (Figure 3). The specific value of each factor corresponded to the corresponding score, and the total score corresponded to 30-day mortality. The nomogram model was internally validated using the bootstrap repeated sampling method 1,000 times. The consistency index C-index was used to test the model discrimination, the nomogram C-index: 0.886, AIC: 167.584. A value close to 1 indicates that the predictive performance of the model is better. Model calibration: The consistency between the predicted probability and the actual probability is evaluated by drawing a calibration curve. The higher the overlap between the fitting curve and the standard curve, the better the fitting degree (Figure 4). Using decision curve analysis (DCA) of the survival data to evaluate the clinical effectiveness, the modeling curves were all above the two extreme curves, indicating good clinical outcomes (Figure 5).

**Figure 6:**
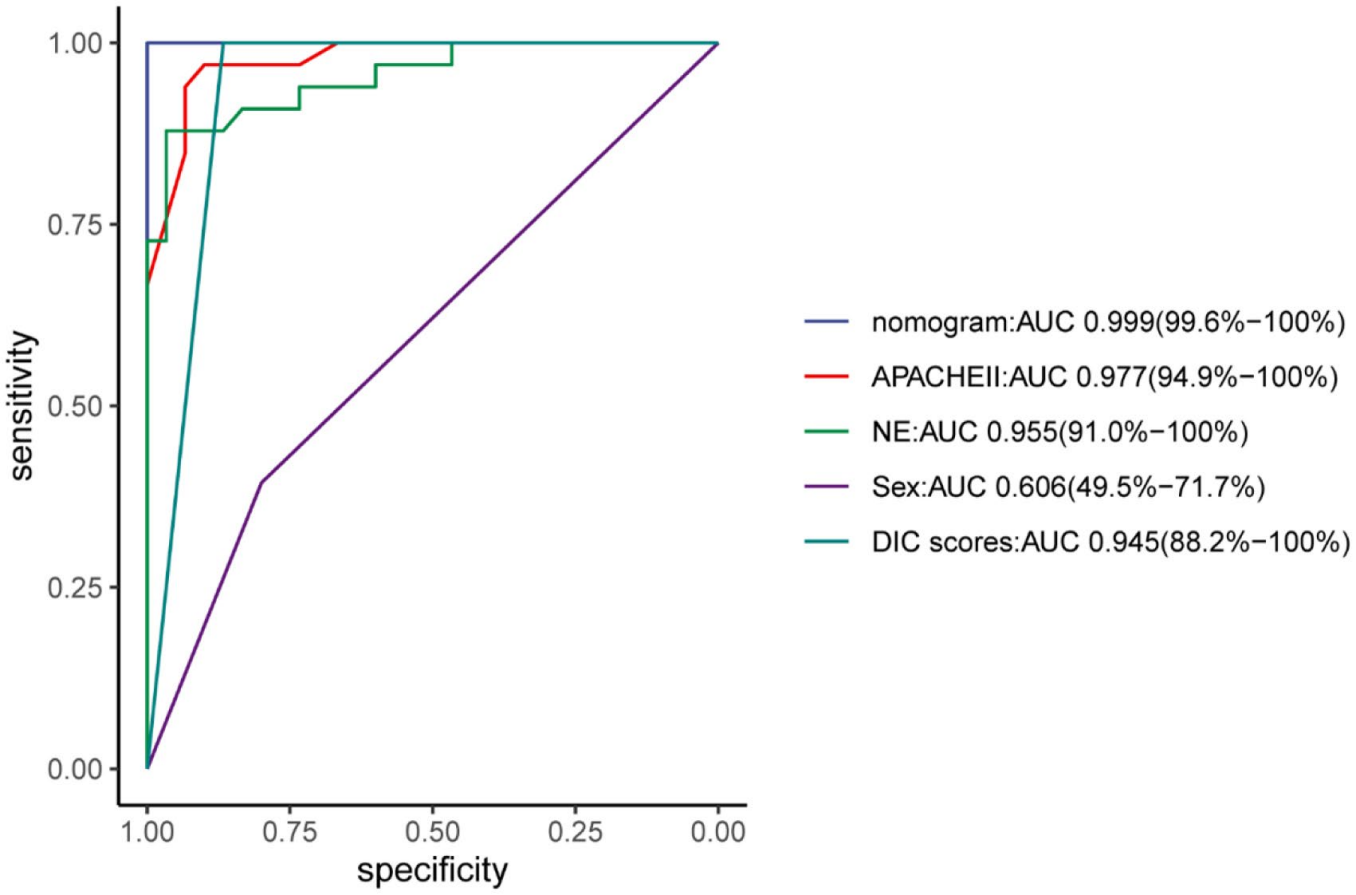
ROC curve.

The nomogram demonstrated good specificity for predicting 30-day survival of ECMO patients. The AUC value of the nomogram was 0.999 (95% CI: 0.96, 1.00), when compared with the NE, Sex, APACHE II and DIC scores, displayed an area under the receiver operating characteristic (AUROC) that was higher in both sets (Figure 6).

**Figure 7:**
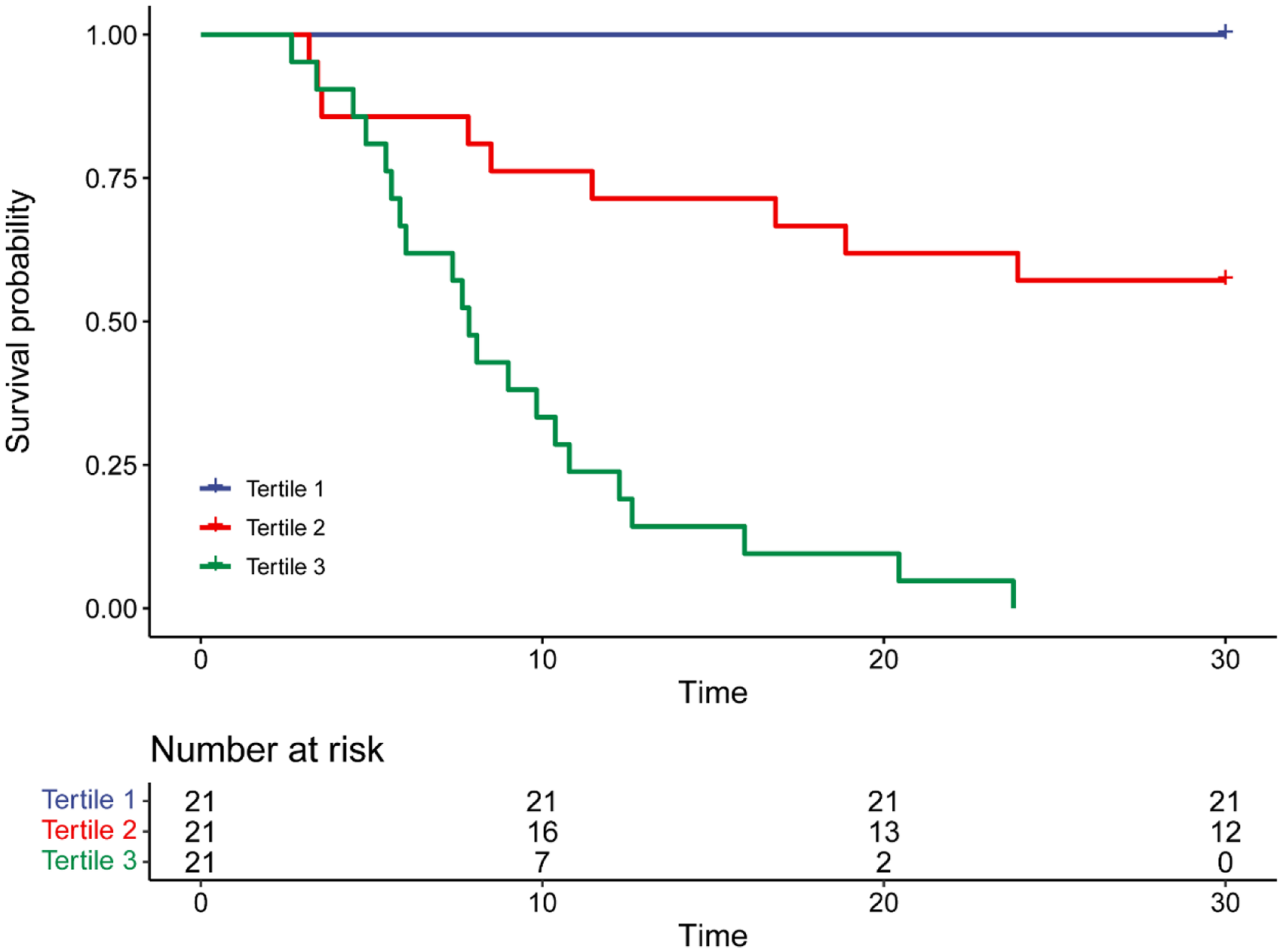
Kaplan-Meier curves are stratified by quartiles of predicted probability.

To further assess the discriminative ability of the model and the predicted probability of the 30-day survival, Kaplan–Meier curves were stratified by the tertile of the predicted probability calculated from the nomograms (Figure 7). Patients with the lowest predicted 30-day survival (tertile 3**)** had a worst outcome (0.00%) than patients in tertile 1 and 2 (100.00 and 57.14%) *(p* < 0.001). Compared with actual survival based on Kaplan–Meier tables, the 10-day survival predicted by the nomogram revealed good estimations of 100.00, 76.19 and 33.33% in tertile 1, 2, 3, respectively (*p* < 0.001). Compared with actual survival based on Kaplan–Meier tables, the 20-day survival rate predicted by the nomogram revealed good estimations of 100.00, 61.90 and 9.52% in tertile 1, 2, 3, respectively (*p* < 0.001) (Figure 7).

## Discussion

In recent years, with the improvement of ECMO equipment and materials, as well as the advancement of technology and the enrichment of clinical experience, the indications for ECMO have been gradually relaxed, and the number of ECMO cases for acute cardiopulmonary failure has increased significantly (14, 15). Despite various challenges, ECMO is considered an important treatment modality (16, 17), but ECMO occupied large amounts of medical resources and greatly increases economic costs. If certain indicators can predict the prognosis of patients before ECMO treatment, it will formulate targeted treatment plans and made a decision whether or not accepted ECMO.

Some studies have shown that the severity of diseases in men and women is similar and even more severe in men, but women admitted to the Department of Critical Care Medicine (18–22). Few studies have demonstrated that the sex affects the survival of critical patients. Although some researchers thought there was no difference in short-term mortality (23, 24), others found that women, especially those over 50 years old, had higher mortality (21). In our study, the Sex was used as an indicator after 30-days survival (HR 3.345, *p* = 0.03).

The APACHE scoring system is widely used in critically ill patients (18). The APACHE II is the most commonly used severity-of-disease scoring system (25) and as a prediction indicator of critical illness mortality and has good prediction capabilities (26). We found that the AUC of the APACHE II before ECMO initiation was 0.977 (94.9–100.00%), can be used as an independent factor for predicting the survival of patients undergoing different types of ECMO.

The DIC scoring system is established based on conventional laboratory tests (27). According to the International Society on Thrombosis and Hemostasis (ISTH) DIC criteria 2018, the optimal cut-off value is 4 (28). All the subjects in our study were accompanied with systemic inflammatory response syndrome (SIRS), which is a key factor in DIC pathogenesis (29). The ISTH DIC score (2018) is a potent tool for predicting mortality and can be applied to non-septic ICU populations (30). In this study, the mortality risk gradually increased as the DIC score increased whether within or greater than 4.

Some studies have shown that the dose of norepinephrine is related to the prognosis (31), and been confirmed in animal models (32). We all knew that the dose of norepinephrine is related to the prognosis of patients with septic shock (33), but there are currently no reports about relationship between the dose of norepinephrine and the mortality of ECMO patients. Our research used the average daily dose of norepinephrine to predict mortality, and the ROC curve area was 0.955 (95.00–100.00%). In this study, the dose of norepinephrine can be a forward indicator of prognosis.

This study aims to establish a predictive model of survival in ECMO patients who can guide subsequent treatment in conjunction with a line diagram and decision-making curve to choose the best plan. The line diagram can provide a more personalized way to provide prognostic information that affects 30-day survival. First, this model can be quantified with simple, clinically applicable terms. Second, it can be used to compare several different models. The line diagram shows good discerning ability, predicting that the prognosis of the 30-day survival is 0.906, the nomogram C-Index: 0.886, AIC: 167.584. The modeling curve was above the two extreme curves, and the net gain was >0, indicating that the prognostic line diagram model has certain clinical guidance for evaluating and predicting 30-day survival.

Several limitations include the following: 1) The confounding factors can occur with the inclusion of each variable, which can affect the results because this study is retrospective. 2) Our study only contains parameters related to the first day in the ICU, and it might have been better to have dynamic, continuous observational analysis data on indicators during the ICU stay. 3) Our database is only a single-center study. In the future, multicenter research can be performed in different countries and regions with different economic levels, and external databases can be used for validation. These are endeavors we will pursue in the future.

## Conclusion

The APACHE II and SOFA score before ECMO initiation, MAP, DIC score and average daily dose of NE were independent risk factors for 30-day survival. The 30-day prognosis line diagram prediction model provides a reference for individual therapy. The number of patients included in this study is limited, and it is also a single-center study. In the future, analyze the correlation maybe need more samples and multi-centers research.

## Data availability statement

The raw data supporting the conclusions of this article will be made available by the authors, without undue reservation.

## Ethics statement

The demographic, physiological and hospital-outcome data were obtained from electronic medical records (EMRs). This retrospective study did not modify the existing diagnostic or therapeutic strategies. This study was approved by the Ethics Committee of the First Affiliated Hospital of Anhui Medical University and granted this study exemption from informed consent due to its retrospective nature. Ethics Approval Number: Kuai-An the First Affiliated Hospital of Medical Sciences-P2021-04-17.

## Author contributions

LC and NL designed and drafted the paper. LC, YZ, CY, HR, MS, and NL were involved in the clinical care and management of the patients. CZ and HZ analyzed the data. All authors approved the final manuscript as submitted and agreed to accountable for all aspects of the work.

## Funding

This work was supported by the Anhui Province Natural Fund, China (1808085MH228).

## Conflict of interest

The authors declare that the research was conducted in the absence of any commercial or financial relationships that could be construed as a potential conflict of interest.

## Publisher’s note

All claims expressed in this article are solely those of the authors and do not necessarily represent those of their affiliated organizations, or those of the publisher, the editors and the reviewers. Any product that may be evaluated in this article, or claim that may be made by its manufacturer, is not guaranteed or endorsed by the publisher.

## Supplementary material

The Supplementary material for this article can be found online at: https://www.frontiersin.org/articles/10.3389/fmed.2023.1062918/full#supplementary-material
