# Supplementary material for: Exploration of a nomogram prediction model of 30-day survival in adult ECMO patients
Source: Front Med (Lausanne). 2023 Feb 28;10:1062918. doi: 10.3389/fmed.2023.1062918 (PMC10011074; doi:10.3389/fmed.2023.1062918)
Supplement: Supplementary file 1 [file Table_1.pdf]

Supplementary table 1. Missing data in the two groups.

Parameters (n, %)	Survival (n=33)	Non-survival (n=30)
PT(s)	10(30.30%)	14(46.67%)
APTT(s)	10(30.30%)	14(46.67%)
CKMB(U/L)	13(39.39%)	16(53.33%)
cTn-I (ng/ml)	12(36.36%)	16(53.33%)
PCT (ng/ml)	13(39.39%)	14(46.67%)
BUN (mmol/l)	10(30.30%)	12(40.00%)
sCr (mmol/l)	10(30.30%)	12(40.00%)
TBIL (mmol/l)	10(30.30%)	13(43.33%)
ALT (U/L)	10(30.30%)	13(43.33%)
AST (U/L)	10(30.30%)	13(43.33%)
PLT (10^9/L)	9(27.27%)	14(46.67%)
pH	11(33.33%)	18(60.00%)
PO_2(mmHg)	11(33.33%)	18(60.00%)
PCO_2(mmHg)	11(33.33%)	18(60.00%)
HCO_3^-(mmol/l)	11(33.33%)	18(60.00%)

PT, prothrombin time; APTT: activated partial thromboplastin time; CKMB: creatine kinase isoenzymes; cTn-I, cardiac troponin I; PCT, procalcitonin; BUN, Blood Urea Nitrogen; sCr, serum creatinine; TBIL, total bilirubin; ALT: alanine aminotransferase; AST: aspartate aminotransferase; PLT: platelet; PO_2: Arterial partial pressure of oxygen;PCO_2, Arterial blood carbon dioxide partial pressure; HCO_3^-, bicarbonate;
