# Supplementary material for: Exploration of a nomogram prediction model of 30-day survival in adult ECMO patients
Source: Front Med (Lausanne). 2023 Feb 28;10:1062918. doi: 10.3389/fmed.2023.1062918 (PMC10011074; doi:10.3389/fmed.2023.1062918)
Supplement: Supplementary file 2 [file Table_2.pdf]

Supplementary table 2. Clinical characteristics of patients between VA-ECMO and VV-ECMO groups

Variable	Total (n=63)	VA-ECMO (n=28	VV-ECMO (n=35)	$t/Z/x^2$ value	*P*-value
Age (year)	53.02±16.02	53.54±15.28	52.60±16.79	0.229	0.820
Sex(male)	44(69.84%)	20(71.43%)	24(68.57%)	0.001	0.976
Height (cm)	167.68±7.50	167.07±7.50	168.17±7.58	0.575	0.567
Weight (kg)	65.95±9.38	65.50±8.53	66.31±9.83	0.340	0.375
Patient sources					
Inside-hospital (n)	27(42.86%)	12(42.85%)	15(42.86%)	0.000	1.000
Peripheral hospitals (n)	36(57.14%)	16(57.14%)	20(57.14%)	0.000	1.000
Comorbidity (n, %)					
Hypertension (n)	16(25.40%)	9(32.14%)	7(20.00%)	0.655	0.419
Diabetes(n)	7(11.11%)	3(10.71%)	4(11.43%)	0.000	1.000
Solid tumors(n)	9(14.29%)	2(7.14%)	7(20.00%)	1.181	0.277
Chronic respiratory disease (n)	4(6.35%)	2(7.14%)	2(5.71%)	0.000	1.000
Chronic cardiac disease (n)	12(19.05%)	9(32.14%)	3(8.57%)	4.181	0.041
Renal diseases (n)	4(6.35%)	1(3.57%)	3(8.57%)	0.083	0.773
Thyroid disease (n)	1(1.59%)	1(3.57%)	0(0%)	0.013	0.910
Nervous system diseases (n)	3(4.76%)	1(3.57%)	2(5.71%)	0.000	1.000
Autoimmune Disease (n)	2(3.17%)	0(0%)	2(5.71%)	0.316	0.574
Temperature (°C)	37.27±0.76	37.39±0.85	37.18±0.69	-1.078	0.285
HR (n)	110.95±20.00	110.82±27.92	111.06±30.25	0.032	0.975
MAP (mmHg)	67.20±12.82	64.59±14.57	69.28±10.99	1.457	0.150
CVP (cmH2O)	14.22±4.15	14.96±3.25	13.63±4.71	-1.276	0.207
Laboratory parameters					
PT(s)	21.74(11.50,82.30)	20.50(17.60,24.90)	19.95(14.73,18.65)	-2.909	0.004
APTT(s)	49.03(27.50,140.60)	45.20(37.00,60.70)	43.50(37.28,52.60)	-0.126	0.899
CKMB(U/L)	117.40(3.00,1176.00)	51.00(13.00,120.00）	18.00(6.00,40.00）	-1.995	0.046
cTn-I (ng/ml)	3.706(0.001,30.00)	0.17(0.00,2.35)	0.12(0.008,0.58)	-0.632	0.653
PCT (ng/ml)	18.85(0.06,169.00)	3.19(0.55,6.39)	5.21(0.58,22.94)	-0.623	0.639
BUN (mmol/l)	12.44(2.60,28.00)	14.18(8.90,16.90)	8.54 (6.75,12.86)	-2.301	0.021
sCr (mmol/l)	156.51(35.50,366.40)	193.40(116.80,267.90)	83.25(61.83,144.40)	-2.719	0.007
TBIL (mmol/l)	25.34(6.50,91.00)	23.40(21.00,36.40)	15.70(8.56,21.92)	-3.074	0.002
ALT (U/L)	457.00(7.00,3743.00)	362.50(67.25,1648.75)	32.00(23.50,46.50)	-4.539	0.000
AST (U/L)	738.54(39.00,696.00)	626.00(87.50,1846.50)	45.00(37.50,78.50)	-3.417	0.001
PLT (10^9/L)	166.63(26.0,429.0)	144.00(84.00,185.00)	175.00(135.00,237.50)	-1.598	0.110

pH	7.27(6.87,7.54)	7.32(7.16,7.47)	7.20(7.16,7.41)	-0.726	0.468	
PO_2(mmHg)	99.27(25.30,440.00）	71.40(61.00,159.13)	63.00(51.75,103.08)	-1.501	0.133	
PCO_2(mmHg)	45.32(19.00,150.00）	35.00(28.35,42.23)	54.00(36.75,60.58)	-2.779	0.005	
HCO_3^-(mmol/l)	21.00(16.45,27.00）	20.00(17.25,26.38)	22.00(14.90,27.60)	-0.685	0.493	
PO2/FIO2	82.50(56.20,192.67)	171.72 (83.09,253.67)	70.80(50.00,90.30)	-3.389	0.001	
Lac	3.80(1.85,7.30)	4.95(2.97,8.60)	2.68(1.51,5.10)	-2.414	0.016	
Steroid (n)	28(44.44%)	11(39.29%)	17(48.57%)	0.232	0.630	
CPR (n)	8(12.70%)	7(25.00%)	1(2.86%)	5.028	0.025	
The daily average dose of NE (ug/kg/min)	1.28±0.74	1.3[illegible]±0.7[illegible]	1.20[illegible]0.55	-1.01[illegible]±0.3[illegible]		2.145
APACHE II	24.00(7.00,44.00)	23.00(8.00,43.00)	24.00(6.00,44.00)	-0.615	0.513	
SOFA	11.00(5.00,19.00)	11.00(6.00,21.00)	12.00(5.00,19.00)	-0.653	0.502	
DIC	3.00(1.00,4.00)	2.50(1.00,4.00)	3.00(0.00,4.00)	-0.056	0.955	
ECMO duration(hour)	158.38(107.875,239.00)	153.00(96.00,199.00)	160.75(114.00,275.00)	-1.092	0.275	
total hospital stay(hour)	528.00(288.00,744.00)	552.00(288.00,724.50)	528.00(293.00,784.00)	-0.180	0.857	
ICU stay(hour)	360.00(264.00,620.00)	360.00(264.00,615.00)	360.00(253.00,672.00)	-0.242	0.809	
ECMO weaning (n)	46(73.02%)	18(64.29%)	28(80.00%)	1.936	0.164	
CRRT (n)[16]	25(39.68%)	11(39.29%)	14(40.00%)	0.000	1.000	
MV duration(hour)	292.50(169.00,472.00)	247.50(173.65,513.75)	303.00(158.60,422.70)	-0.021	0.983	
Complication						
Thrombosis (n)	5(7.94%)	2(7.14%)	3(8.57%)	0.000	1.000	
Bleeding (n)	6(9.52%)	3(10.71%)	3(8.57%)	0.000	1.000	
pneumothorax (n)	2(3.17%)	0(0%)	2(5.71%)	0.316	0.574	
HAIs(n)[3]	33(52.38%)	13(46.43%)	20(57.14%)	0.351	0.554	

ECMO, Extracorporeal membrane oxygenation; HR, heart rate; MAP, mean arterial pressure; CVP, central venous pressure; PT, prothrombin time; APTT: activated partial thromboplastin time; CKMB: creatine kinase isoenzymes; cTn-I, cardiac troponin I; PCT, procalcitonin; BUN, Blood Urea Nitrogen; sCr, serum creatinine; TBIL, total bilirubin; ALT: alanine aminotransferase; AST: aspartate aminotransferase; PLT: platelet; PO_2: Arterial partial pressure of oxygen;PCO_2, Arterial blood carbon dioxide partial pressure; HCO_3^-, bicarbonate; PO_2/FiO_2, oxygenation index; Lac, lactate; V-A, venoarterial; V-V, Venovenous; CPR, cardiopulmonary resuscitation`; NE , norepinephrine; APACHE II, acute physiology and chronic health evaluation II; SOFA, sequential organ failure assessment; DIC, disseminated intravascular coagulation. CRRT, Continuous Renal Replacement Therapy; ICU, intensive care unit; MV, mechanical ventilation; HAIs, hospital acquired infections.
